# Application of visible and near-infrared spectroscopy to classification of *Miscanthus* species

**DOI:** 10.1371/journal.pone.0171360

**Published:** 2017-04-03

**Authors:** Xiaoli Jin, Xiaoling Chen, Liang Xiao, Chunhai Shi, Liang Chen, Bin Yu, Zili Yi, Ji Hye Yoo, Kweon Heo, Chang Yeon Yu, Toshihiko Yamada, Erik J. Sacks, Junhua Peng

**Affiliations:** 1 Department of Agronomy & The Key Laboratory of Crop Germplasm Resource of Zhejiang Province, Zhejiang University, Hangzhou, China; 2 Hunan Provincial Key Laboratory for Germplasm Innovation and Utilization of Crop, Hunan Agricultural University, Hunan Changsha, China; 3 Wuhan Botanical Garden, Chinese Academy of Sciences, Wuhan, Hubei, China; 4 Wuhan Junxiu Horticultural Science and Technology Co., Ltd. Wuhan, Hubei, China; 5 Kangwon National University, Chuncheon, Gangwon, South Korea; 6 Field Science Center for Northern Biosphere, Hokkaido University, Sapporo, Hokkaido, Japan; 7 Department of Crop Sciences, University of Illinois, Urbana-Champaign, Urbana, Illinois, United States of America; 8 Life Science and Technology Center, China National Seed Group Co., Ltd., Wuhan, Hubei, China; Agricultural University of Athens, GREECE

## Abstract

The feasibility of visible and near infrared (NIR) spectroscopy as tool to classify *Miscanthus* samples was explored in this study. Three types of *Miscanthus* plants, namely, *M*. *sinensis*, *M*. *sacchariflorus* and *M*. *fIoridulus*, were analyzed using a NIR spectrophotometer. Several classification models based on the NIR spectra data were developed using line discriminated analysis (LDA), partial least squares (PLS), least squares support vector machine regression (LSSVR), radial basis function (RBF) and neural network (NN). The principal component analysis (PCA) presented rough classification with overlapping samples, while the models of Line_LSSVR, RBF_LSSVR and RBF_NN presented almost same calibration and validation results. Due to the higher speed of Line_LSSVR than RBF_LSSVR and RBF_NN, we selected the line_LSSVR model as a representative. In our study, the model based on line_LSSVR showed higher accuracy than LDA and PLS models. The total correct classification rates of 87.79 and 96.51% were observed based on LDA and PLS model in the testing set, respectively, while the line_LSSVR showed 99.42% of total correct classification rate. Meanwhile, the lin_LSSVR model in the testing set showed correct classification rate of 100, 100 and 96.77% for *M*. *sinensis*, *M*. *sacchariflorus* and *M*. *fIoridulus*, respectively. The lin_LSSVR model assigned 99.42% of samples to the right groups, except one *M*. *fIoridulus* sample. The results demonstrated that NIR spectra combined with a preliminary morphological classification could be an effective and reliable procedure for the classification of *Miscanthus* species.

## Introduction

The giant grasses, some species in *Miscanthus*, have high potential of biomass productivity and could be used as a feedstock of renewable energy. Recently, *Miscanthus sacchariflorus*, *M*. *sinensis* and *M*. *fIoridulus* were proved to the most potential biomass grass species. Xi and Jeźowski presented systematic positions of these species, *M*. *sacchariflorus*, *M*. *sinensis* and *M*. *fIoridulus* belong to Subtrib. *Saccharinae* [1]. These three species generally grow in the similar environment; even grow together [1, 2]. Meanwhile, they demonstrate similar morphology, so the identification of *Miscanthus* varieties has to been achieved by morphological examination, field investigation, observation of cultivated accessions and statistical analysis [15]. In order to classify the species, the detailed morphological traits, e.g., plant height, stem diameter near base; culm number, auricles, panicle, glumes, root types and so on, in the whole growth period, have to be observed and recorded [1].

Up to date, several methods have been tried to distinguish the *Miscanthus* species. The DNA content of diploid was tested as 4.37 ±0.02 pg/2C in *M*. *lutarioriparius*, 4.37 ± 0.01 pg/2C in *M*. *sacchariflorus*, and 5.37 ± 0.03 pg/2C in *M*. *sinensis*, respectively, by flow cytometry of nuclei in 36 populations [3]. In their study, the genome size of *M*. *fIoridulus* was not assessed. Thus, *M*. *sacchariflorus*, *M*. *sinensis* and *M*. *fIoridulus* can’t be well distinguished. *Miscanthus* species showed high levels of genetic variation within and between species [4]. There is also spontaneous triploid species in the nature. Overall, these methods are time-consuming, laborious, expensive, or require highly skilled taxonomy experts. So far, there is no effective method to distinguish *M*. *sacchariflorus*, *M*. *sinensis* and *M*. *fIoridulus*. Taking these into account, it is very important for us to develop a rapid, inexpensive and efficient approach for distinguishing *Miscanthus* species.

Near-infrared (NIR) spectroscopy is a very efficient method for high-throughput screening of plant materials for their chemical characteristics. It provides rapid, nondestructive, low-cost and environment-friendly measurements. Based on the correlation among the vibration properties of organic molecule chemical bonds and their interactions with infrared radiation, NIR spectrum has been applied to the qualitative and quantitative analyses of biological and non-biological materials such as food, agriculture, textile and pharmaceutical fields and so on [5–7]. Furthermore, NIR spectroscopy has been used in the classification of materials. Using NIR, Wu et al. constructed models for stalk soluble sugars, bagasse hydrolyzed sugars, and three major cell wall polymers in bioenergy sweet sorghum [8]. The NIR spectroscopy was also applied to predict the methane yield at 29 days, cellulose, acid detergent fiber, neutral detergent fiber and crude protein of forbs and grass-clover mixture. The best prediction models were obtained for methane yield at 29 days, cellulose, acid detergent fiber, neutral detergent fiber and crude protein (R^2^ > 0.9) [9]. Using NIR spectra and PLS multivariate analysis, the calibration models were built to predict the feedstock composition and the release and yield of soluble carbohydrates generated [10]. NIR spectroscopy was also used in *Miscanthus* to predict the lignocellulosic components, biomass digestibility, moisture, calorific value, ash and carbon content [6, 11,12]. Zhao et al. used NIR spectroscopy to clarify wheat geographical origins [13]. According to different floral origins of Chinese honey samples, the feasibility of NIR spectroscopy and multivariate analysis as tools to classify samples was explored. An artificial neural network (ANN) model resulted in total correct classification rates of 90.9% and 89.3% for the calibration and validation sets [14].

Therefore, NIR spectroscopy combined with a classification technique could be a feasible approach for the classification of materials. The major objective of this current study is to apply visible and NIR spectroscopy to species identification of the important biomass grass plant, *Miscanthus*.

## Materials and methods

### *Miscanthus* samples and classification using the classical botanical method

A total of 517 *Miscanthus* accessions originated from China, Korea, Japan, Russia (Table 1) were planted in the *Miscanthus* fields in Zhejiang, Hunan and Hubei provinces. Of these materials, 141 *M*. *sacchariflorus*, 92 *M*. *sinensis* and 26 *M*. *fIoridulus* were collected from a *Miscanthus* garden in Zhejiang province (Zhuji, China, E120°09.441’, N29°49.509’). Meanwhile, we collected 30 *M*. *sacchariflorus*, 30 *M*. *sinensis* and 65 *M*. *fIoridulus* accessions in Hunan province (Changsha, China, E113°04’08.4”, N28°11’14.6’). The remaining samples were collected in Hubei province (Wuhan, China, E113°04’08.4”, N28°11’14.6”) (Table 1). Before sample collection, the materials were distinguished using the classical taxonomy in botany [1, 15]. Fresh leaves of each accession were collected and stored at 4°C before scanning.

**Table 1:** Information of *Miscanthus* species, amount, locations in the sampling regions.

Growth location	Species	Origin	No. of samples	Latitude	Longitude	Elevation (m)
Zhejiang Province	*M*. *sinensis*	China, Japan, Korea	92	N29°49.509’	E120°09.441’	56
	*M*. *sacchariflorus*	China, Japan, Korea, Russia	141			
	*M*. *fIoridulus*	China	26			
Hubei Province	*M*. *sinensis*	China	30	N30°09.138”	E114°17.160’	34
	*M*. *sacchariflorus*	China	100			
	*M*. *fIoridulus*	China	3			
Hunan Province	*M*. *sinensis*	China	30	N28°11.146’	E113°04.084’	47
	*M*. *sacchariflorus*	China	30			
	*M*. *fIoridulus*	China	65			

### Visible and near infrared measurements

All samples were scanned in transmission mode (400–2500 nm) with an interval of 2 nm using a scanning monochromator FOSS NIRSystems 6500 (FOSS NIRSystems, Silver Spring, MD, USA) in reflectance mode. Spectral data were collected using Vision software (version 3.5.0.0). Thirty-two scans were performed for each sample. From the 517 samples, 345 were randomly selected for the calibration (S1 Table), and the remaining 172 were used for the validation test (S2 Table). The spectrum of each sample was recorded three times.

### Spectral data pre-treatment

In order to reduce the systematic noise and strengthen contribution of the chemical composition to the spectral signal, specific spectral preprocessing methods were applied to the mean spectral data of each sample. The preprocessing methods including wavelet transform (WT) smoothing with segment size of 3, area normalization, spectroscopic transformation, multiplicative scatter correction (MSC), the first derivative of the calibration spectra calculated with 3 gaps of data points, baseline and standard normal variance with de-trending (SNV-D) were used in this study, respectively. The pretreatments were carried out according to the users’ manual of the Unscrambler V9.5 (CAMO PROCESS AS, Oslo, Norway). The effect of pretreatment was assessed by naked eyes and partial least-squares regression (PLS).

### Multivariate data analysis

To classify *Miscanthus* species, the principal component analysis (PCA), linear discriminant analysis (LDA) and partial least-squares (PLS) models were developed using full cross validation in the present study. PCA is a statistical procedure that uses an orthogonal transformation to convert a set of observations of possibly correlated variables into a set of values of linearly uncorrelated variables, principal components (PC). PCA was performed as a tool to extract the main information from the multivariate data set in this study. Each sample had a score along each PC used for further modeling instead of spectral data. In addition, the clustering between different groups could be examined preliminarily by plotting PCs.

LDA was carried out using the SPSS 16.0 software package (SPSS Inc., Chicago, IL). It yields a number of orthogonal linear discriminant functions that allow the samples to be classified into one or another category. By calculating the squared Mahalanobis distance of a sample from their gravity centers of the considered groups, the samples close to the gravity center of were clustered into one group. LDA was carried out for the *Miscanthus* from different regions. Every sample set was randomly split up into two groups, with 345 samples as the calibration set and the remaining 172 as the test set. The results were analyzed in terms of the percentage of correct classification of samples.

PLS was conducted using the Unscrambler V9.5 (CAMO PROCESS AS, Oslo, Norway). The PLS is a bilinear modeling method for relationship between a set of independent spectral variables (X) and a single dependent variable (Y). PLS algorithm was used as the regression method to develop classification models. PLS regression uses group number as the dependent variable Y and the wavelengths as the independent X variables. Group membership of a new unknown sample is determined by its predicted value with PLS. A sample is considered to be correctly categorized, if the predicted value lies on the two sides of the assigned values. In this study, samples from a the specific region under examination in each PLS model were assigned a *Miscanthus* species value of 1, 2 and 3, and threshold value was ±0.5. For models of least square support vector regression (LSSVR) and radial basis function neural network (RBF_NN), the same *Miscanthus* species value of 1, 2 and 3, and threshold value of ±0.5 were also assigned.

The LSSVR was carried out based on the LS-SVM toolbox of MATLAB (Version 7.8.0.347, The MathWorks. Inc US). In order to evaluate classification performance of the models and to reduce risk of the overly optimistic results, the models were tested through cross-validation (CV) and by predicting the results for independent sets of samples (test sets). Moreover, the models were optimized by computing a random subset cross-validation with three iterations.

The RBF_NN is a type of nonlinear neural network, which is used to solve several types of classification and regression problems. The theory of RBF_NN has been described extensively [16]. All calculations of RBF_NN were implemented based on the Neural Networks toolbox of MATLAB (Version 7.8.0.347, The MathWorks. Inc US). A model with a low standard error of calibration (SEC), a low standard error of prediction (SEP), and a high *r* was considered as good model [5]. Meanwhile, the residual prediction deviation (RPD), defined as the standard deviation (SD) of the samples’ reference values divided by SEC for the NIR spectroscopy calibrations, was a good index to evaluate the quality of regression models. A relatively high RPD value indicates that the model is able to reliably predict the chemical composition. In order to establish an optimal network structure with a low SEP and a short convergence time, different networks are often trained under a wide range of parameter settings, including learning rates, momentums, and different numbers of input nodes and hidden nodes [16, 17].

## Results

### Classical classification

Before we collected the leaf samples, all the 517 *Miscanthus* accessions were distinguished using the classical procedures of taxonomy in botany [1,15]. The characters used to distinguish samples were summarized in S3 Table. Morphological traits of *Miscanthus* species including habit, rhizome, adventitious roots, branches of culms etc., were observed. The 517 *Miscanthus* accessions were successfully classified into three species, 271 of *M*. *sacchariflorus*, 152 of *M*. *sinensis* and 94 of *M*. *fIoridulus* (Table 1, S4 Table).

### NIR analysis

**Fig 1:**
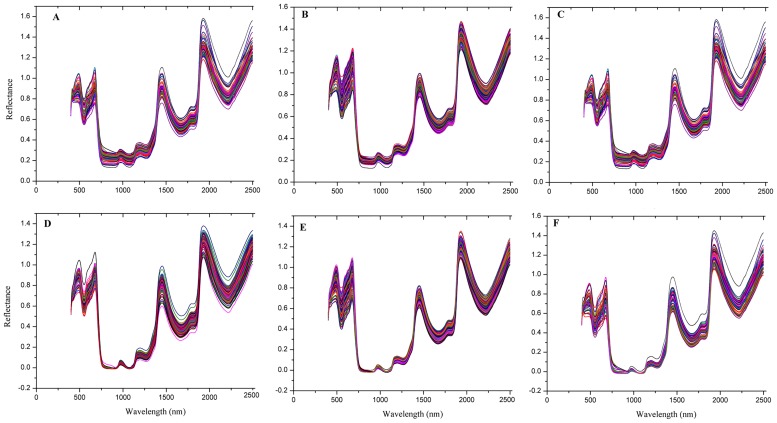
Raw spectra and the spectra with baseline treatment of *Miscanthus* varieties. A-C, raw spectra of *M*. *sinensis*, *M*. *sacchariflorus* and *M*. *fIoridulus*; D-F, the spectra with baseline treatment of *M*. *sinensis*, *M*. *sacchariflorus* and *M*. *fIoridulus*.

The wavelength range from 400 to 2500 nm with an interval of 2 nm was used in our study. The main peaks of *Miscanthus* were marked in Fig 1. Fig 1A, 1B and 1C show the original spectra of the three *Miscanthus* species. The trend of the spectral curves is quite similar and there are overlaps among the three species. No outliers could be identified directly by the naked eye. Therefore, further data treatment is needed and then the latent features of the spectra will be applied for classification. In order to find out the best pretreatment, the effect of pretreatments was evaluated one by one (Table 2). Of them, the baseline treatment showed significant improvement. Thus, we only used the spectral data with baseline pre-treatment for further analysis. Seven peaks in Fig 1A to 1F were found, respectively, while the peaks in Fig 1D, 1E and 1F treated with baseline were sharper and clearer than those in Fig 1A, 1B and 1C. The results indicated that the data with baseline treatment might include more obvious NIR information than the other pre-treatments.

**Table 2:** *Miscanthus* classification based on the PLS models with different pretreatments in the full spectral range.

Pretreatment	*M*. *sinensis*			*M*. *sacchariflorus*			*M*. *fIoridulus*			Total		
	Total No.	Right No.	CC (%)	Total No.	Right No.	CC (%)	Total No.	Right No.	CC (%)	Total No.	Right No.	CC (%)
Original	51	48	94.12	90	88	97.78	31	29	93.55	172	165	95.93
Smoothing		48	94.12		88	97.78		29	93.55		165	95.93
Normalization		48	94.12		88	97.78		29	93.55		165	95.93
Spectroscopic transformation		46	90.20		86	95.56		26	83.87		158	91.86
MSC		46	90.20		26	28.89		27	87.10		99	57.56
1^st^ derivative		46	90.20		87	96.67		27	87.10		160	93.02
Baseline		48	94.12		89	98.89		29	93.55		166	96.51
SNV-D		46	90.20		87	96.67		27	87.10		160	93.02

CC%: correct classifications

As shown in Fig 1, two strong peaks with wavelength of 488 nm and 676 nm located in the visible spectra range, which could be related to -C = O, while one weak peak with wavelength of 974 nm and a strong peak with wavelength of 1930 nm are assigned to the 2nd overtone and combination of free -OH, respectively [18]. The band at approximately 1198 nm of wavelength is attributable to C–H stretching (the 2nd overtone) [19,20]. The signal peaks at wavelengths of 1452 nm and 1794 nm are ascribed to the combination of C-H stretching, CH3 and CH2 (the 1st overtone), respectively [20,21].

### PCA analysis

**Fig 2:**
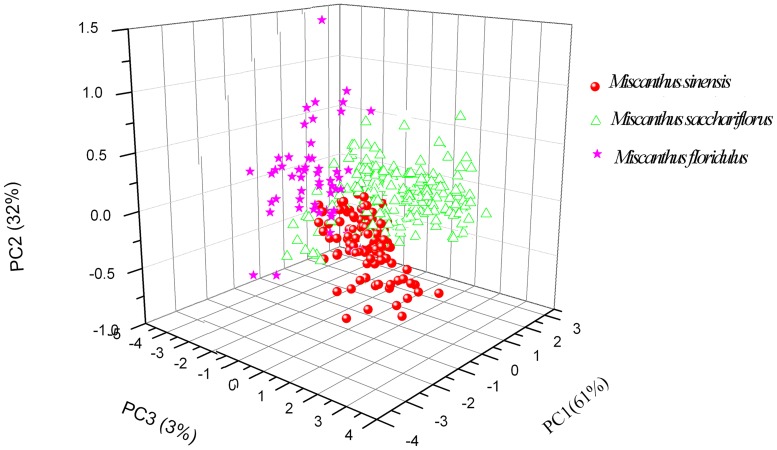
The first three PC score plot of *Miscanthus* samples.

PCA was performed on the 517 *Miscanthus* samples, in order to figure out the possible classification of *Miscanthus* species using the visible and NIR spetra. The first three PCs account for 96% of the variation in the NIR spectra of *Miscanthus* samples, and the score plot of the first three PCs was showed in Fig 2. A rough separation among three *Miscanthus* species was observed and some overlaps were also present. Therefore, further chemometric methods including LDA, PLS and LS-SVR were used to construct models for the *Miscanthus* classification.

### LDA analysis

The 517 *Miscanthus* samples collected from Zhejiang, Hubei and Hunan provinces (Table 1) were split into a calibration set including 101 *M*. *sinensis*, 181 *M*. *sacchariflorus* and 63 *M*. *fIoridulus* accessions and a validation set including 51 *M*. *sinensis*, 90 *M*. *sacchariflorus* and 31 *M*. *fIoridulus* accessions, respectively (Table 2). The PCA models were developed for *M*. *sinensis*, *M*. *sacchariflorus* and *M*. *fIoridulus* accessions, respectively. Subsequently, the PCA scores were used as input data for LDA. LDA was performed on the first five PCs from PCA, which accounted for 99% variation of the spectral data. LDA results for NIR analysis are shown in Table 2. In terms of *Miscanthus* samples, rate of correct classification of 88.24%, 86.67% and 87.10% were achieved for *M*. *sinensis*, *M*. *sacchariflorus* and *M*. *fIoridulus*, respectively, while the F score for these *Miscanthus* species were 0.88, 0.88 and 0.84, respectively (Table 2). LDA models were developed using specific pairs from different *Miscanthus* species. This resulted in 95% correct classification between *M*. *sinensis* and *M*. *sacchariflorus*, 93.6% correct classification between *M*. *sacchariflorus* and *M*. *fIoridulus*, and 94.6% between *M*. *sinensis* and *M*. *fIoridulus*. These classification results indicated that there are specific chemical components and morphological traits in *Miscanthus* samples from different species.

### PLS analysis

PLS results for *Miscanthus* samples are shown in Table 2. The rates of correct classification for *M*. *sinensis*, *M*. *sacchariflorus* and *M*. *fIoridulus* are 94.12, 98.33 and 93.55%, respectively, while the F scores of them were 0.97, 097 and 0.95, respectively. Of them, the PLS model for *M*. *sacchariflorus* with higher rate of correct classification and f score was obviously better than that for other two *Miscanthus* species. The results indicated that there were differences of NIR spectra among *Miscanthus* species. The 96.51% of total correct classification rate was achieved, higher than the first two ways of PCA and PLS analysis.

### LS-SVM and RBF_NN analysis

In our study, calibration models for Lin_LSSVR, RBF_LSSVR and RBF_NN arithmetics were developed. The modeling parameters should be optimized before LSSVR model is developed. Two parameters, including γ and the δ^2^ are very important in the RBF kernel function. The γ is a regularization parameter, which determines the tradeoff between the structural and empirical risk minimization. The δ^2^ is the kernel width parameter and plays an important role in improving the generalization performance of the LSSVR model. The combination of the two-step grid search approach and the ten-fold cross-validation was utilized for global optimization of these parameters in this study. The initial values of γ and δ^2^ in the RBF_LSSVR model were both set as 2. The range of γ and δ^2^ was 0.001–100,000 and 0.001–500,000, respectively. The logarithmic transformation was employed in the search plane due to the large magnitude in the investigated ranges of these parameters. The optimal value of γ and δ^2^ was obtained as 4.4590 e + 003 and 3.3941 e + 004, respectively. The optimal parameters of the LSSVR model were set to the same values as for Lin_LSSVR, RBF_LSSVR and RBF_NN models.

**Fig 3:**
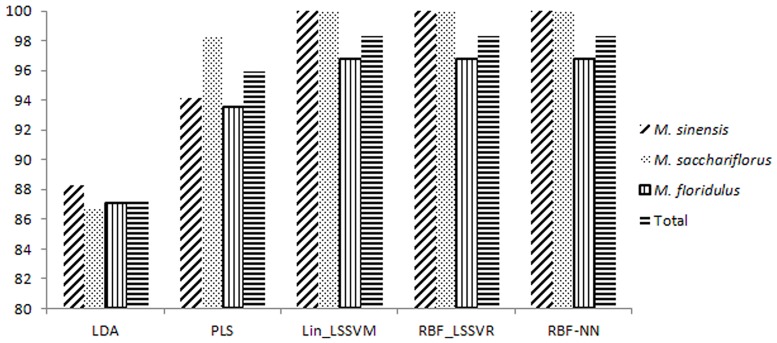
The bar chart of five calculation models for *M*. *sinensis*, *M*. *sacchariflorus* and *M*. *fIoridulus*.

In order to optimize the network, an appropriate learning rate and different numbers of input nodes and of hidden layer neurons were all examined. In this study, a three-layer model was achieved at last, with the sigmoid transfer function. The output layer was set as one neuron. After multi-training, the optimal number of nodes in the hidden layer and the learning rate were defined as 12 and 0.2, respectively. At the optimal parameter settings, the RBF_ANN model was developed. The goal error was set as 0.001. The correct classification rate and f scores in the three models were almost the same (Table 3). Due to the higher computation speed, we preferred using the Lin_LSSVR model in the classification of *Miscanthus* species other than RBF_LSSVR and RBF_NN models. The correct classification rate in Lin_LSSVR model for *M*. *sinensis*, *M*. *sacchariflorus*, *M*. *fIoridulus* in calibration set was 98.86%, 99.54% and 93.65%, respectively, while correct classification rate for these three groups in the validation set were 100%, 100% and 96.77%, respectively (Table 4). The model could assign 99.42% of the samples to the right groups, except for that one sample of *M*. *fIoridulus* was wrongly grouped. Compared with LDA and PLS, Lin_LSSVR model showed the highest accuracy (Table 3; Fig 3).

**Table 3:** Classification of *Miscanthus* accessions using LDA, PLS, LS-SVM, RBF_LSSVR and RBF_NN.

Species	Method	Total No.	Right No.	CC (%)	Precision (%)	Recall rate(%)	F score
*M*. *sinensis*	LDA	51	45	88.24	88.24	88.24	0.88
	PLS		48	94.12	100	94.12	0.97
	Lin-LSSVM		51	100	100	100	1
	RBF_LSSVR		51	100	100	100	1
	RBF_NN		51	100	100	100	1
*M*. *sacchariflorus*	LDA	90	78	86.67	88.64	86.67	0.88
	PLS		89	98.89	94.68	98.89	0.97
	Lin-LSSVM		90	100	98.90	100	0.99
	RBF_LSSVR		90	100	98.90	100	0.99
	RBF_NN		90	100	98.90	100	0.99
*M*. *fIoridulus*	LDA	31	27	87.10	81.82	87.10	0.84
	PLS		29	93.55	96.67	93.55	0.95
	Lin-LSSVM		30	96.77	100	96.77	0.98
	RBF_LSSVR		30	96.77	100	96.77	0.98
	RBF_NN		30	96.77	100	96.77	0.98
Total	LDA	172	150	87.21	87.21	87.21	0.87
	PLS		166	96.51	96.51	96.51	0.97
	Lin-LSSVM		171	99.42	99.42	99.42	0.98
	RBF_LSSVR		171	99.42	99.42	99.42	0.98
	RBF_NN		171	99.42	99.42	99.42	0.98

CC: correct classifications

**Table 4:** Calibration and prediction results for the classification of the *Miscanthus* species according to the model developed by Lin_LSSVM.

Species	CC%	
	Calibration	Validation
*M*. *sinensis*	98.86	100
*M*. *sacchariflorus*	99.54	100
*M*. *fIoridulus*	93.65	96.77
Total	98.13	99.42

CC%: correct classifications

## Discussion

Up to date, there is no report on classification of *Miscanthus* species using NIR spectroscopy. In previous studies, the classification of *Miscanthus* species was mainly based on the morphological traits [1, 15]. According to the classical botanical classification standard (S3 Table), we examined and recorded all the morphological traits of samples during whole growth period. Based on the morphological observations and with the assistance of a professional plant taxonomist, Professor Yi Ren of Shanxi normal university, we unambiguously classified the 517 *Miscanthus* samples into three species, *M*. *sacchariflorus*, *M*. *sinensis* and *M*. *fIoridulus* (Table 1, S3 Table). So far, there is still no simple and easy way to distinguish the three species of *Miscanthus*. It is necessary for us to develop a rapid, cost-effective and user-friendly technique for discriminating the *Miscanthus* varieties.

Recently, models using the near-infrared spectroscopy were developed for measurement of chemical components and classification in plants. Several models were established to figure out the geographical origin of corn distillers dried grains with solubles, amazonian tree species and hazelnut, wheat and the different floral origins of Chinese honey samples by NIR spectroscopy [13–14,22–25]. Leaves are complex assemblies of organic compounds and, because of this, may be expected to exhibit different spectral responses. *M*. *sacchariflorus*, *M*. *sinensis* and *M*. *fIoridulus* may show different intensity and position of the absorption bands in leaves. These differences may be related to several factors possibly associated with the compounds, hydrogen bonds, and crystallinity. As reported in the previous studies, the leaf size and shape [26, 27], phenology [28], photosynthesis and water-use strategies [29], and even the complex cell wall components in *Miscanthus* could be variable and thus result in the variation of the NIR spectra. Therefore, we used the visible and NIR spectroscopy of leaves in the present study to distinguish plant species of *Miscanthus*.

In our study, five calibration models were built, namely LDA, PLS, Lin_LSSVR, RBF_LSSVR and RBF_NN arithmetics. As shown in Fig 2, we could not clearly distinguish the experimental samples using LDA, because of the partial overlaps among the species, and 88.24, 86.67 and 87.10% of the samples were assigned correctly to the *M*. *sinensis*, *M*. *sacchariflorus* and *M*. *fIoridulus* group, respectively. When using the PLS model, the rate of correct classification was enhanced to 94.12, 98.33 and 93.55%, respectively, for the three species. There still were obviously wrongly assigned accessions. In order to further improve the grouping accuracy, other three models including Lin_LSSVR, RBF_LSSVR and RBF_NN, were applied. These three models demonstrated good correlation between the expected and the actual values. Only one *M*. *fIoridulus* sample was wrongly grouped. Among the methods of Lin_LSSVR, RBF_LSSVR and RBF_NN, the computation of RBF_LSSVR and RBF_NN are more complicated and spend more time and energy than Lin_LSSVR. Furthermore, the three models, Lin_LSSVR, RBF_LSSVR and RBF_NN showed almost the same accuracy (Fig 3; Table 3). Therefore, the Lin_LSSVR model should be recommended to classification of *Miscanthus* accessions.

Using the Lin_LSSVR model based on NIR spectra of leaves, the samples of *M*. *sinensis*, *M*. *sacchariflorus* and *M*. *fIoridulus* can be unambiguously discriminated in the present study except only one samples was wrongly grouped in the models of Lin_LSSVR, RBF_LSSVR and RBF_NN (Fig 3; Tables 2 & 3). The models of Lin_LSSVR, RBF_LSSVR and RBF_NN showed higher accuracy. Moreover, the cost-efficiency of spectroscopy-based method is high due to the high speed, low cost and ability to achieve successful hits. The method of scanning leaves by NIR spectrophotometer and based on the Lin_LSSVR model is truly a cost-effective approach for sample classification of *Miscanthus* plant.

## Supporting information

S1 Table The data of visible and near-infrared spectroscopy with baseline treatment and classification for the calibration set. (XLSX)

S2 Table The data of visible and near-infrared spectroscopy with baseline treatment and classification for the validation set. (XLSX)

S3 Table GPS data for the Miscanthus accessions examined. (XLS)

S4 Table The classical standards of classification for three *Miscanthus* species. (XLS)
